# Supplementary material for: Supramolecular Interactions of Teixobactin Analogues in the Crystal State
Source: J Org Chem. 2024 Mar 20;89(7):5104–8. doi: 10.1021/acs.joc.3c02617 (PMC11002827; doi:10.1021/acs.joc.3c02617)
Supplement: Supplementary file 1 — jo3c02617_si_001.pdf [file jo3c02617_si_001.pdf]

Supporting information:
Supramolecular Interactions of Teixobactin Analogues in the Crystal State

Hyunjun Yang,[a] Adam G. Kreutzer,[a] and James S. Nowick[a,b,*]

[a] Department of Chemistry, University of California Irvine, Irvine, CA 92697, United States.
[b] Department of Pharmaceutical Sciences, University of California Irvine, Irvine, CA 92697, United States.
E-mail: jsnowick@uci.edu

Table of Contents

Supplementary Tables

Table S1. X-ray crystallographic data collection and processing. S1

Table S2. X-ray crystallographic structure solution and refinement. S2

Figure S1. HPLC trace of *N*-Me-D-Gln_4,Lys_{10}-teixobactin. S3

Figure S2. Mass spectrum of *N*-Me-D-Gln_4,Lys_{10}-teixobactin. S4

Supplementary Tables

Table S1. X-ray crystallographic data collection and processing.

Diffraction source	ALS 8.2.2 beamline	ALS 8.2.2 beamline
Wavelength (Å)	2.0663	0.9997
Temperature (K)	100	100
Detector	3×3 CCD array (ADSC Q315R)	3×3 CCD array (ADSC Q315R)
Rotation range per image (°)	1	1
Total rotation range (°)	720	720
Space group	*P*43	*P*43
a, *b*, *c* (Å)	25.86, 25.86, 109.50	24.88, 24.88, 106.32
α, β, γ (°)	90, 90, 90	90, 90, 90
Resolution range (Å)	2.022–27.40	1.148–24.88
Total No. of reflections	4147	20644
Completeness (%)	99.75	99.91
$\langle I/\sigma(I)\rangle$	45.72	24.09
Overall *B* factor from Wilson plot (Å^2)	30.61	14.35

Table S2. Structure solution and refinement.

Resolution range (Å)	27.38–2.40 (2.486–2.40)	24.88–1.50 (1.554–1.50)
Completeness (%)	99.75 (100)	99.90 (100)
σ cutoff	$F > 1.36\sigma(F)$	$F > 1.36\sigma(F)$
No. of reflections, working set	2805 (268)	10318 (1055)
No. of reflections, test set	282 (26)	1030 (106)
Final R_{cryst}	0.2297 (0.2912)	0.1544 (0.2188)
Final R_{free}	0.2494 (0.4508)	0.1831 (0.2643)
Peptide	8	8
Water	70	74
Chloride	9	14
Ligand		Pentaethylene glycol dimethyl ether
Bonds (Å)	0.011	0.023
Angles (°)	1.44	2.30
Average B factors (Å^2)	30.61	20.41
Ramachandran plot		
Most favoured (%)	87.5	100
Allowed (%)	12.5	0

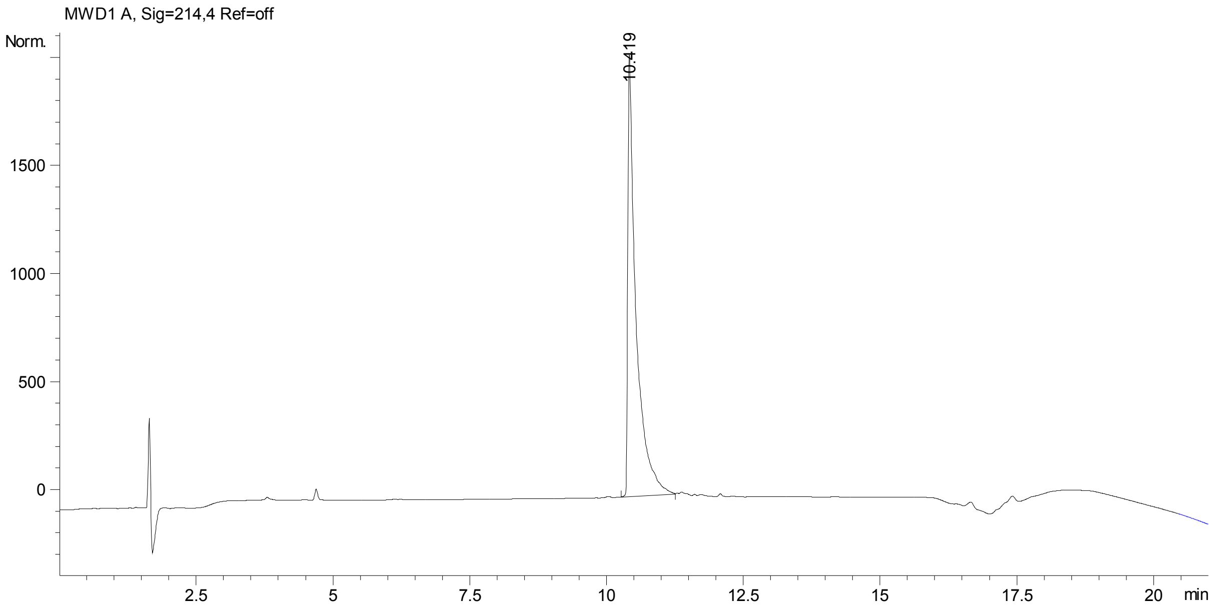

Figure S1. HPLC trace of *N*-Me-D-Gln4,Lys10-teixobactin.

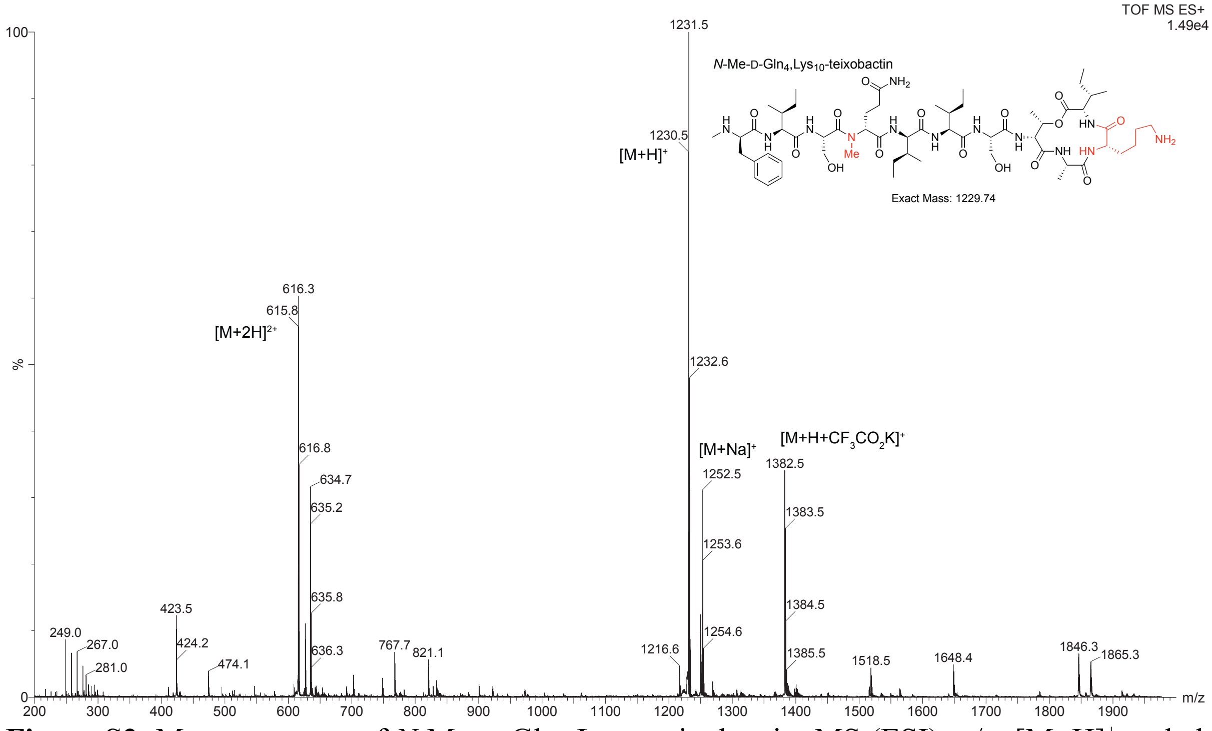

Figure S2. Mass spectrum of *N*-Me-D-Gln4,Lys10-teixobactin. MS (ESI) m/z: $[M+H]^+$: calcd for $C_{59}H_{100}N_{13}O_{15}$ 1230.75; Found 1230.5. Waters (Micromass) LCT Premier was used to collect ESI-MS in the positive mode and direct flow-injection was used for rapid analysis.
